# Waterfowl: Potential Environmental Reservoirs of the Chytrid Fungus *Batrachochytrium dendrobatidis*

**DOI:** 10.1371/journal.pone.0035038

**Published:** 2012-04-13

**Authors:** An Garmyn, Pascale Van Rooij, Frank Pasmans, Tom Hellebuyck, Wim Van Den Broeck, Freddy Haesebrouck, An Martel

**Affiliations:** 1 Department of Pathology, Bacteriology and Avian Diseases, Faculty of Veterinary Medicine, Ghent University, Merelbeke, Belgium; 2 Department of Morphology, Faculty of Veterinary Medicine, Ghent University, Merelbeke, Belgium; Imperial College Faculty of Medicine, United Kingdom

## Abstract

Infections with *Batrachochytrium dendrobatidis* (*B. dendrobatidis*), the causal agent of chytridiomycosis, have been shown to play an important role in the decline of amphibians worldwide. Spread of the fungus is poorly understood. Bird movement might possibly contribute to the spread of *B. dendrobatidis* in the environment. Therefore, 397 wild geese in Belgium were screened for presence of *B. dendrobatidis* on their toes using real-time quantitative PCR (qPCR). In addition, chemotaxis towards, adhesion, survival after desiccation and proliferation of *B. dendrobatidis* on keratinous toe scales from waterfowl were examined *in vitro*. qPCR revealed that 76 geese (15%) were positive for *B. dendrobatidis*. Results of the *in vitro* tests showed that *B. dendrobatidis* is attracted to the keratinous toes of aquatic birds on which they can adhere and even proliferate. However, desiccation is poorly tolerated. This suggests waterfowl are potential environmental reservoirs for *B. dendrobatidis*.

## Introduction

Last decades significant declines in amphibians have been observed which makes these species currently the most threatened vertebrate class on the planet [1]. Several of these declines have been linked to the presence of a pathogenic chytrid fungus *B. dendrobatidis*, the causal agent of chytridiomycosis [2], [3]. Many members of the chytrid family are parasitic, infecting plants, algae, protists and invertebrates [4]. *B. dendrobatidis* is the only known member of the chytrid family that infects vertebrate hosts. The fungus colonizes the keratinized layers of the amphibian epidermis or the keratinized anuran larval mouthparts [5], [6]. In clinical infections this is associated with hyperplasia and hyperkeratosis resulting in disruption of the skin's osmoregulatory function, deshydratation, electrolyte imbalance and mortality [6], [7]. Despite previous attemps to identify potential environmental reservoirs or vector hosts, the spread of this pathogen or mechanisms that allow its persistence in the environment are currently poorly understood. Many members of the chytrid family have, beside a parasitic lifestage, the ability to develop and reproduce saprophytically [4]. Also for *B. dendrobatidis* evidence for such saprophytic lifestages, utilizing non-amphibian organic materials as nutrients, are suggested. Under laboratory conditions, for instance, *B. dendrobatidis* can be cultured on tryptone agar without any keratin [5]. In addition, the fungus can survive up to 3 months in moist sterile river sand and grows on sterile feathers, dead algae and arthropod exoskeletons [8], [9]. Such reservoirs might allow persistence of the fungus outside its natural host. Unfortunately, outside the lab environmental reservoirs for *B. dendrobatidis* have not yet been demonstrated [10]. In addition, the spread of the disease could be facilitated by vector hosts transmitting the infection to susceptible species. Good evidence exist that multiple asymptomatically infected amphibian species are implicated in translocation of the fungus to new habitats and in naïve populations [11], [12], [13]. In addition, a recent study describes experimental *B. dendrobatidis* infections in nematodes (*Caenorhabditis elegans*) [14]. This might indicate the possibility of alternative host species.

Since waterfowl and amphibian assemblages often co-occur, their role as potential reservoir was assessed in this study. For this, a large population of wild geese in Belgium was screened for the presence of *B. dendrobatidis* on their toes using real-time quantitative PCR (qPCR). To study the interaction of *B. dendrobatidis* with the avian foot into more detail, chemotaxis towards and adhesion of *B. dendrobatidis* on keratinous toe tissue were examined. Also proliferation and viability after desiccation of the fungus on avian toe scales were tested.

## Results

### *B. dendrobatidis* is highly prevalent on geese toes

Results of the qPCR assays showed that 15% (76/497) of the geese tested positive for presence of *B. dendrobatidis*. All 6 wildlife areas examined were affected and both *Branta canadensis* (68/456) and *Anser anser domesticus* (8/41) were carriers of *B. dendrobatidis* (**Table 1** **.**). The number of genomic equivalents (GE) detected on the swabs varied between 0.1 GE and 469 GE. Overall, mean numbers were 19.2 GE/swab.

**Table 1:** Prevalence of *B. dendrobatidis* on geese toes.

Location	Positive geese/sampled geese		Mean GE/positive sample (SD) [range]
	*Branta canadensis*	*Anser anser domesticus*	
Oudenaarde	3/32	1/17	27.2 (51.8) [0.3–104.9]
Wachtebeke	16/203	0/1	1.1 (1.28) [0.1–5.3]
Berlare	13/34	7/23	11.0 (25.51) [0.2–113.1]
Drongen	31/51	-	35.6 (86.2) [468.6–0.3]
De Pinte	2/25	-	1.2 (0.17) [1.0–1.3]
Destelbergen	3/11	-	3.60 (3.46) [0.10–7.02]

A total of 397 wild geese, originating from 6 wildlife areas in East Flanders (Belgium) were sampled. For each location, the number of geese positive for *B. dendrobatidis* per sampled geese and the mean genomic equivalents (GE) per positive sample are illustrated. The standard deviation and range of the genomic equivalents across the positive samples are shown between brackets.

### *B. dendrobatidis* is attracted to keratinous scales of goose toes

Positive migration of *B. dendrobatidis* towards toe scales of a goose was observed. After 45 minutes zoospore counts in the squares adjacent to the toe scale were 11, 25 and 35 (mean count = 24; standard deviation (SD) = 12). After 90 minutes zoospore counts were increased to 26, 52 and 68 (mean count = 49; SD = 21). At the opposite side of the counting chamber zoospores were never observed. In the three control assays with cellulose acetate filters as attractant only 0, 1 and 2 zoospores were counted after 45 minutes (mean count = 1; SD = 1). After 90 minutes zoospore counts were 1, 2 and 2 (mean count = 2; SD = 1). Again, zoospores were not observed at the opposite side of the counting chamber. After statistical analysis the p-values related to the t-test for repeated measures, resulted three times in p<0.01 indicating a significant difference comparing the means of the two experimental groups at the 0.05 significance level. This determines that toe scales have a significant effect as attractant, compared to the cellulose acetate filter.

### *B. dendrobatidis* adheres on duck and swan toe scales and proliferates on goose toe scales

**Figure 1:**
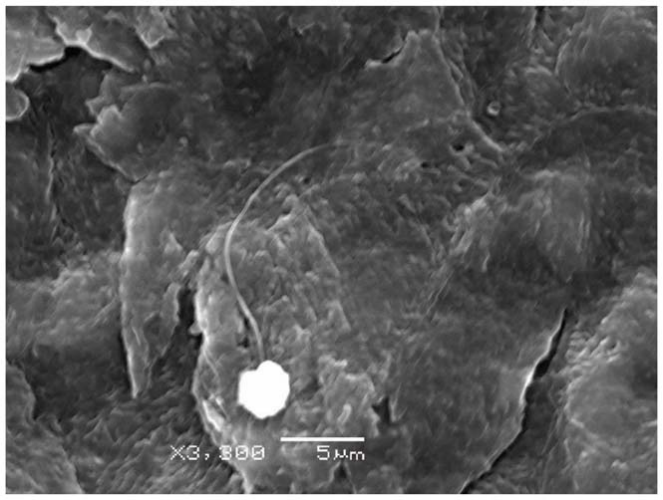
Adhering *B. dendrobatidis* zoospore on swan toe webbings. Scanning electron microscopic image of a *B. dendrobatidis* zoospore adhering on toe webbings of a swan 30 minutes after incubation.

**Figure 2:**
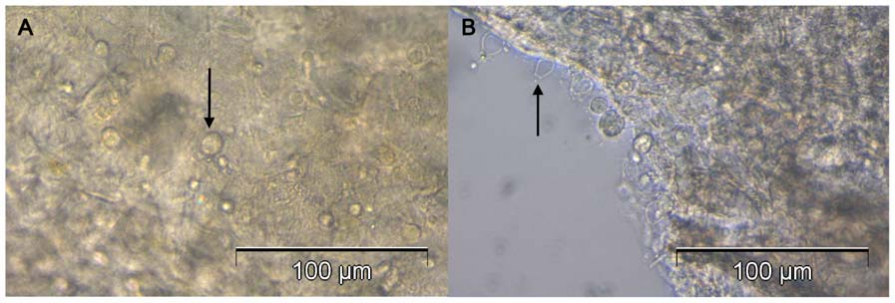
Colonization of *B. dendrobatidis* on duck toe squamae. Light micrograph of *B. dendrobatidis* colonization on Barbary duck toe squamae, showing A: abundant sporangia (arrow) present upon the surface of the keratinous squamae; B: post-discharge sporangia (arrow); magnification 400×.

*B. dendrobatidis* zoospores adhered to the surface of swan and duck toe webbings within 30 minutes of incubation (**Figure 1**). During the *in vitro* growth test on goose toe scales, *B. dendrobatidis* zoospores had encysted and adhered to the surfaces after 24 hours. Motile zoospores were absent. From day 4 on, numerous motile zoospores were present and a clear colonization of the scale surfaces with numerous sporangia and post-discharge sporangia was observed (**Figure 2**). Motile zoospores were still observed until the end of the experiment (14 days of incubation). Besides chytrid sporangia associated with the toe scales, no sporangia were observed elsewhere in the wells. After 0 days of incubation, zoospores counts in every well were zero. After 14 days of incubation, zoospore counts in the three replicates were 880, 1350 and 1350 zoospores/well. The results of the qPCR analysis of the inoculated scales did not show an increase in GE after 14 days of incubation. Mean GE numbers were on average 1.99×10^3^ GE (SD 0.46) and 1.68×10^3^ GE (SD 0.70) after 0 days and 14 days of incubation, respectively.

### *B. dendrobatidis* zoospores on toe scales can survive a desiccation period of 30 minutes

When inoculated onto toe scales, both zoospores and zoosporangia proved viable after a desiccation period of 0 minutes (controls). Growth of *B. dendrobatidis* was observed in every well when distilled water was added after the desiccation period had finished. Also after 30 minutes of desiccation, the zoospores were still viable in all three replicates. From the three-day-old zoosporangia 2 positive cultures were obtained. From the five-day-old zoosporangia only 1 culture was positive. *B. dendrobatidis* zoospores and zoosporangia were not able to survive a desiccation period of 60 minutes. Results of the desiccation tests are given in **table 2**.

**Table 2:** Survival rate of *B. dendrobatidis* on duck toes after desiccation.

	Number of growth positive wells/total number of wells		
Desiccation period	Zoospores	3 day old sporangia	5 day old sporangia
0 min	3/3	3/3	3/3
30 min	3/3	2/3	1/3
60 min	0/3	0/3	0/3

Viability of zoospores and zoosporangia after different desiccation periods was assessed in distilled water. The detection of active zoospores after desiccation was regarded as a positive assay. When motile zoospores could not be detected the assay was regarded negative. Three replicates were run.

## Discussion

In this study, wild geese were screened for the presence of *B. dendrobatidis* on their toes using qPCR and the interaction of the fungus with the avian foot was studied *in vitro*. The results of the qPCR screening prove that chytrid DNA is present on geese toes. Previously, it has been hypothesized that birds might play a role in the dissemination of *B. dendrobatidis* in the environment [10], but the presence of this fungus on birds had not yet been demonstrated. Unfortunately, qPCR only detects pathogen DNA. The results of the *in vitro* tests, however, demonstrate that *B. dendrobatidis* is actively attracted to, adheres and proliferates on toe scales. After colonization, *B. dendrobatidis* encysts and develops sporangia. Already after 4 days, discharge tubes are formed and new zoospores released. This finding provides evidence that *B. dendrobatidis* is able to reproduce as a saprobe. Such saprophytic life stage, although common in other members of the chytrid family [4], has never been documented for *B. dendrobatidis* before. After 2 weeks of incubation, the number of zoospores was increased up to 1320 zoospores/well. Considering the high inoculation dose (10^6^ zoospores), these relative low zoospore counts could explain that replication was too limited to result in an increase of GE's after qPCR analysis. However, since motile zoospores were continuously present in the growth assays from 4 days onwards, this provides evidence that feet of waterfowl are suitable for successful maintenance of *B. dendrobatidis* for at least a fortnight. This implies that birds may act as non-amphibian reservoirs of the fungus, allowing persistence of the fungus outside its natural host. Additionally, *B. dendrobatidis* is able to survive a drying period of 30 minutes. In this time span, geese could fly up to 30 km [15]. This means that, when attached to the avian feet, the fungus might survive when geese move to a pond nearby.

On the other hand, to see things in a proper perspective, two comments are to be made. First, for the *in vitro* growth test, toe scales were autoclaved to prevent overgrowth from residual microbial flora. Although no macroscopically visible changes were noticed, this procedure might have altered the structure of the tissue. In addition, elimination of resident microbiota may affect *B. dendrobatidis'* survival. This might render the test results less indicative for the situation in the wild. Secondly, due to different habitat preferences, the chance of direct contact between geese and amphibians might be rather limited. However, the wildlife areas sampled in this study contain amphibian communities with *Pelophylax kl. esculentus*, *Bufo Bufo*, *Lissotriton vulgaris* and *Ichthyosaura alpestris* [16]. In general, geese prefer large wetlands, lakes and rivers [17] whereas many European amphibians prefer ponds. In conclusion, although qPCR does not give information on the viability of the zoospores detected, the prevalence of *B. dendrobatidis* on the toes of geese and the high number of GE sometimes found here, together with our findings that *B. dendrobatidis* is attracted to, adheres and proliferates on keratinous avian toes indicates that aquatic birds may act as non-amphibian reservoirs of *B. dendrobatidis*.

## Materials and Methods

### Screening wild geese for *Batrachochytrium dendrobatidis*

Wild geese (356 *Branta canadensis* and 41 wild *Anser anser domesticus*) were caught in 6 different wildlife areas in East Flanders (Belgium) as part of an eradication programme [18], transported to the Faculty of Veterinary Medicine (UGhent) and euthanized by intravenous embutramid injection (T61, Intervet, Belgium). Immediately after euthanasia samples for *B. dendrobatidis* detection were taken by swabbing the plantar side of the toes of the geese using cotton-tipped swabs (Copan Diagnostics Inc., Corona, CA). After sampling, the carcasses of the geese were destroyed conform health regulations. Subsequently DNA from the swabs was extracted and qPCR assays were performed as described previously [19]. The study was conform regulations of the Ethical and Animal Welfare Committee of the Faculty of Veterinary Medicine of Ghent University.

### *B. dendrobatidis* strain and collection of keratinous tissue for *in vitro* use

*In vitro* inoculations were carried out with zoospores of the *B. dendrobatidis* strain IA042, kindly provided by Dr. T. Garner and Dr. M. Fisher. The strain was isolated from a dead *Alytes obstetricans* involved in a mass mortality event (Ibon Acherito, Spanish Pyrenees) and belongs to the Global panzootic lineage [20]. For inoculation, strain IA042 was cultured in tryptone/gelatine hydrolysate/lactose (TGhL) broth, in 25 cm2 cell culture flasks, at 20°C for 5 days. A 2 ml-aliquot of a 5-day old broth culture was transferred onto a TGhL agar plate, and incubated for 5–7 days at 20°C. Zoospores were collected by flooding the agar plate with 2 ml of distilled water, and were immediately counted in Lugol's solution by using a haemocytometer and adjusted to the final inoculum concentration.

Avian keratinous toe tissue for the *in vitro* tests were collected from a dead swan (*Cygnus cygnus*), two dead Moscovy ducks (*Cairina moschata*) and a goose (*Anser anser domesticus*) which were brought to the department of Pathology, Bacteriology and Avian diseases (Faculty of Veterinary Medicine, UGhent) for post-mortem examination. The keratinous toe scales of these birds were isolated with a forceps from the rinsed feet and stored at room temperature until further use. From both animals the toe webs were excised and stored at −20°C until further use.

### Chemotaxis of *B. dendrobatidis* towards toe scales

Chemotaxis of *B. dendrobatidis* towards geese toe scales was determined as described previously [21], with minor modifications. Briefly, a 10 µl droplet of distilled water was placed directly on the surface of the counting grid of a Burker counting chamber (Marienfeld, Lauda-Königshofen, Germany). A keratinous toe scale of approximately 5 mm was placed at one side of the grid. After this a cover slip was placed on the counting chamber and 10 µl inoculum (4×10^5^ zoospores/ml) was carefully added at the other side of the grid. For the negative controls, instead of toe scales, 5 mm discs perforated out of sterilized cellulose acetate filters (0.45 µm, Sartorius Stedim, Aubagne, France) and impregnated with distilled water were used. Slides were checked under an inverted microscope (Olympus CKX 41, Hamburg, Germany) to ensure that zoospores initially accumulated just before the grid. The number of motile and immobile zoospores in the squares adjacent to the toe scale and on the other side of the grid were counted after 45 and 90 minutes incubation at 20°C. The assay was carried out in triplicate.

For the statistical analysis a linear mixed effect model for repeated measures was conducted in MLwin with the group as a fixed effect. To compare the two chemotaxis groups, a t-test was used with the alternative hypothesis being that zoospore counts adjacent to toe scales were higher. The tests were performed at a global 5% significance level. Since count data were analysed, a Poisson distributed error was conducted.

### Adhesion and *in vitro* growth of *B. dendrobatidis* on duck and swan toes

The extent of adhesion of *B. dendrobatidis* zoospores to duck and swan toes over a relatively short time span, ranging from 30 minutes to 2 hours was examined. Both toe scales and toe webbings of a swan and a Barbary duck were inoculated in a 24 well plate (Cellstar ®, Greiner Bio-One, Wemmel, Belgium) with 25 µl of a 10^6^ zoospore suspension in distilled water. To each well, 975 µl distilled water was added. Plates were sealed and incubated at 20°C for 30 minutes, 1 or 2 hours. After incubation, the samples were washed three times in distilled water, centrifuged at 1500 rpm at 20°C and fixed in a 2.5% glutaraldehyde, 2% formaldehyde containing HEPES buffer (pH 7.2) for scanning electron microscopy (SEM). The samples were post-fixed in 1% osmium tetroxide (w/v, OsO_4_) in distilled water for 2 h at room temperature and were dried with hexamethyldisilazane (Electron Microscopy Sciences, Hatfield, Pennsylvania, USA) in a fume hood. Finally, scales and toe webbings were mounted on metal bases and platinum sputter-coated (JFC–1300 Auto Fine Coater, JEOL Ltd, Zaventem, Belgium), prior to examination with a JSM-5600LV scanning electron microscope (JEOL Ltd).

In addition, the ability of *B. dendrobatidis* to colonize and grow on geese toe scales was examined. Two geese scales were autoclaved (to prevent overgrowth by residual microbial flora), transferred into the wells of a 96-well plate and inoculated with 200 µl of a 10^6^ zoospore suspension in distilled water. Prior to inoculation the zoospores had been washed 3 times with distilled water and centrifuged at 1500 rpm, at 20°C. Plates were sealed and incubated at 20°C for 24 hours. After this incubation period, scales were rinsed in distilled water to remove non adherent zoospores and transferred into a new well containing 200 µl freshly distilled water. Subsequently, 10 µl of the well contents was placed directly on the surface of the counting grid of a Burker counting chamber (Marienfeld, Lauda-Königshofen, Germany) and zoospore counts were performed. This was done by counting the total surface of the grid. After this, one scale and its corresponding well contents were immediately stored at −20°C. The second scale was incubated for 14 days at 20°C. During incubation, growth (development of sporangia and release of active zoospores) was evaluated daily using inverted microscopy. After 14 days of incubation, a zoospore count was performed as described above after which the scale and their corresponding well contents was stored at −20°C. DNA of the frozen samples was extracted and qPCR assays were performed as described previously [20]. The assay was performed in 3-fold.

### Survival of *B. dendrobatidis* on duck toe scales after desiccation

The viability of *B. dendrobatidis* zoospores and zoosporangia on duck toes after a 0, 30 and 60 minute period of desiccation was assessed. To test the viability of zoospores, toe scales of a Barbary duck were autoclaved and subsequently inoculated in a 24 well-plate with 10 µl inoculum containing 7×10^4^ zoospores in distilled water. Prior to inoculation, zoospores had been washed 3 times in distilled water and centrifuged at 1500 rpm at 20°C. The wells were dried under a laminar flow after which they were incubated at 20°C for 0 (positive control wells), 30 and 60 minutes. After this incubation period, 1 ml of distilled water was added to each well. Plates were sealed and incubated at 20°C. Growth was evaluated during 7 days using inverted microscopy. The detection of active zoospores after desiccation was regarded as a positive assay. When motile zoospores could not be detected the assay was considered negative. The assay was carried out in triplicate.

To test the viability of zoosporangia, autoclaved toe scales were placed in a 24 well-plate and inoculated with 1 ml of a 7×10^4^ zoospores suspension in distilled water. Plates were incubated at 20°C and the zoospores were allowed to develop into zoosporangia on the scales for 3 to 5 days. Scales were then removed and transferred to a new 24 well-plate. The scales were dried under a laminar flow after which they were incubated at 20°C for 0 (positive control wells), 30 and 60 minutes. Subsequently, the assay was performed in triplicate as described above.
